# Genome-Wide Identification of Expansin Genes in Wild Soybean (*Glycine soja*) and Functional Characterization of *Expansin B1* (*GsEXPB1*) in Soybean Hair Root

**DOI:** 10.3390/ijms23105407

**Published:** 2022-05-12

**Authors:** Xu Feng, Cuiting Li, Fumeng He, Yongqing Xu, Li Li, Xue Wang, Qingshan Chen, Fenglan Li

**Affiliations:** 1 College of Life Sciences, Northeast Agricultural University, Harbin 150030, China; 18045043687@163.com (X.F.); licuiting123789@163.com (C.L.); hefumeng@neau.edu.cn (F.H.); doctor_smith@163.com (Y.X.); yibo8818@163.com (L.L.); wang_x2022@126.com (X.W.); 2 Key Laboratory of Soybean Biology of Chinese Education Ministry, Harbin 150030, China; 3 College of Agriculture, Northeast Agricultural University, Harbin 150030, China

**Keywords:** wild soybean, expansin, genome-wide identification, cultivated soybean, salt stress

## Abstract

Wild soybean, the progenitor and close relative of cultivated soybean, has an excellent environmental adaptation ability and abundant resistance genes. Expansins, as a class of cell wall relaxation proteins, have important functions in regulating plant growth and stress resistance. In the present study, we identified a total of 75 members of the expansin family on the basis of recent genomic data published for wild soybean. The predicted results of promoter elements structure showed that wild soybean expansin may be associated with plant hormones, stress responses, and growth. Basal transcriptome data of vegetative organs suggest that the transcription of expansin members has some organ specificity. Meanwhile, the transcripts of some members had strong responses to salt, low temperature and drought stress. We screened and obtained an expansin gene, *GsEXPB1*, which is transcribed specifically in roots and actively responds to salt stress. The results of *A. tumefaciens* transient transfection showed that this protein was localized in the cell wall of onion epidermal cells. We initially analyzed the function of *GsEXPB1* by a soybean hairy root transformation assay and found that overexpression of *GsEXPB1* significantly increased the number of hairy roots, root length, root weight, and the tolerance to salt stress. This research provides a foundation for subsequent studies of expansins in wild soybean.

## 1. Introduction

Soybean (*Glycine max*) is a major source of vegetable oil and plant protein worldwide, and its special nitrogen fixation ability makes it a high-profit crop in rotation systems and intercropping cultivation models [1]. However, the genetic diversity of soybean has gradually decreased during long-term human evolutionary selection and breeding domestication, and its resistance to environmental stress has gradually diminished [2]. Wild soybean (*Glycine soja*) is an ancestral and close relative of cultivated soybean that has long-term survival under natural conditions and shows strong tolerance to abiotic stresses [3]. Mining environmental stress resistance genes in wild soybean and applying them to germplasm improvement in cultivated soybean can not only improve stress resistance and quality but can also effectively solve the problems of a narrow genetic basis and poor environmental adaptation [4]. The introduction of high-quality resistance gene resources from wild species into cultivars is a shortcut to crop variety improvement and has been successful in breeding efforts for a wide range of crops [5,6].

In 1992, expansin was first identified in the hypocotyl of cucumber (*Cucumis sativus*), which relaxes the cell wall of plants with nonenzymatic activity and depends on pH [7]. Expansins are widespread in plants, and members of this family arose from a common ancestor and have formed four subfamilies over the course of evolution, including α-expansin (EXPA), β-expansin (EXPB), expansin-like A (EXLA), and expansin-like B (EXLB) [8]. The proteins of this family are more evolutionarily conserved, with similarity of amino acid sequences between subfamilies typically ranging from 20–25%, usually having two conserved domains, DPPB and CBM63 [9]. Expansin acts directly on the cell wall of plants, and alterations in cell wall morphological structure mediated by expansin are important pathways for plants to undergo growth and adapt to environmental changes [10,11]. Numerous studies have shown that expansins play important functions in regulating seed germination [12,13], root development [14,15], leaf growth [16,17], stem elongation [18], stomatal opening and closing [19,20], flower development [21], fruit ripening [22,23], and seed yield [24,25,26,27]. Meanwhile, expansins play active roles in plant defense against drought [28,29], salt [30,31], low temperature [32,33], and heavy metal stresses [34], and their expression is always regulated by multiple phytohormones [35,36].

Among the molecular breeding of agricultural crops at this stage, promoting plant growth and improving environmental stress resistance is one of the main focuses. Because the function of expansin is compatible with the demand of breeding, related research efforts in a variety of plants have been carried out systematically. Genome-wide identification of expansin genes in food crops, economic crops, vegetables and fruits such as wheat (*Triticum aestivum*) [37], maize (*Zea mays*) [38], soybean [39], tobacco (*Nicotiana tabacum*) [40], peanut (*Arachis hypogaea*) [41], tomato (*Solanum lycopersicum*) [42], and apple (*Malus domestica*) [43] has been successively accomplished. However, the identification and functional studies of the expansin family in wild soybean have not been previously reported. In this study, we systematically identified the expansin family based on recent genomic information of wild soybeans while initially analyzing the function of *GsEXPB1*, a member related to salt stress tolerance. This study provides a reference for further understanding of the characteristics of the wild soybean expansin family and its value in the molecular breeding of cultivated soybean.

## 2. Results

### 2.1. Identification of Expansin Family Members in Wild Soybean

**Figure 1:**
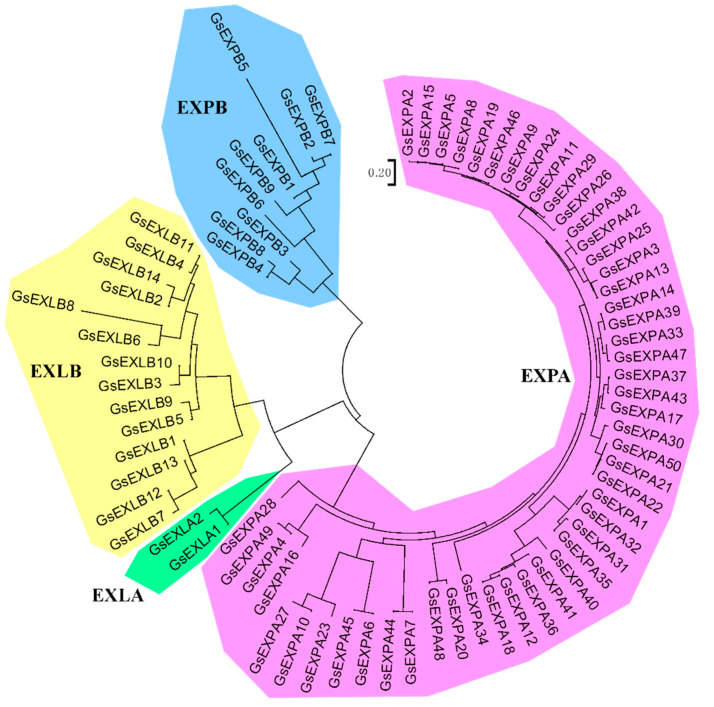
Amino acid phylogenetic tree of wild soybean expansin family members.

After primary screening and secondary identification, we identified 75 members of the expansin family in the wild soybean genome, including 50 members of EXPA, 9 members of EXPB, 2 members of EXLA, and 14 members of EXLB. The construction of the amino acid evolution tree is shown in Figure 1. These expansin proteins range in length from 155–316 amino acids, signal peptide lengths from 18–39 amino acids, isoelectric points from 4.49–9.8, and molecular weights from 16.7–34.3 kDa. Detailed information is presented in Supplementary Table S1.

### 2.2. Gene Structure and Conserved Motifs

**Figure 2:**
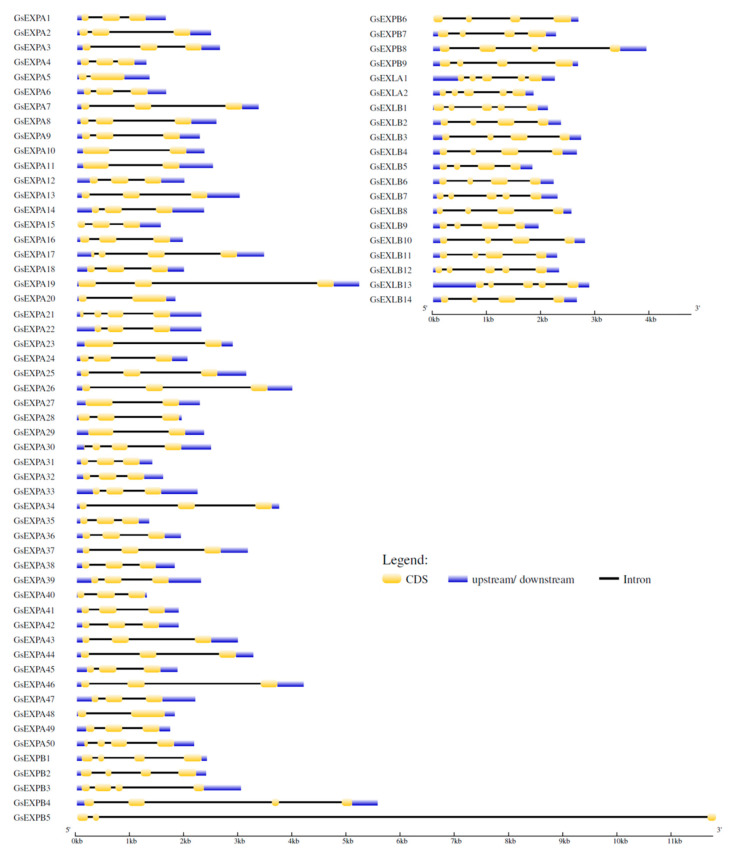
Gene structure analysis results of wild soybean expansin family members.

**Figure 3:**
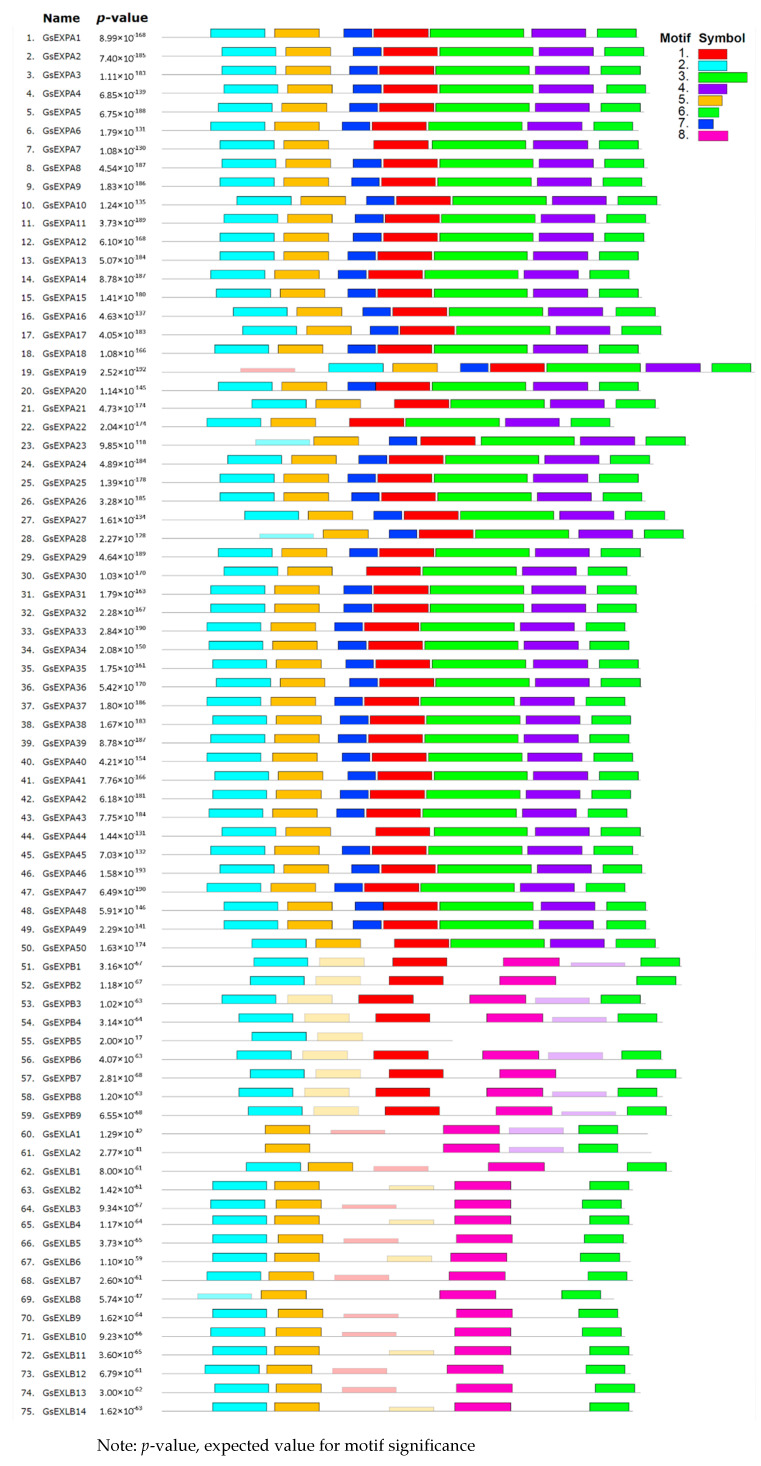
Analysis results of wild soybean expansins conserved motifs. *p* value, expected value for motif significance.

We analyzed the gene structure and conserved motifs of wild soybean expansin family members. The number of introns in these members is 1–4, and the introns of *GsEXPB5* are particularly long, exceeding 11 kb. Members with a larger number of introns are mostly from the EXLA and EXLB families, and EXPA family members always have 1–2 introns (Figure 2). Meanwhile, we identified eight conserved motifs (Figure 3). Motifs 2 and 6 are highly conserved in all expansin proteins, and motifs 3, 4, and 7 are unique to the EXPA family. The EXPA family usually lacks motif 8, whereas motif 1 is hard to find in EXLAs and EXLBs.

### 2.3. Cis-Elements

**Figure 4:**
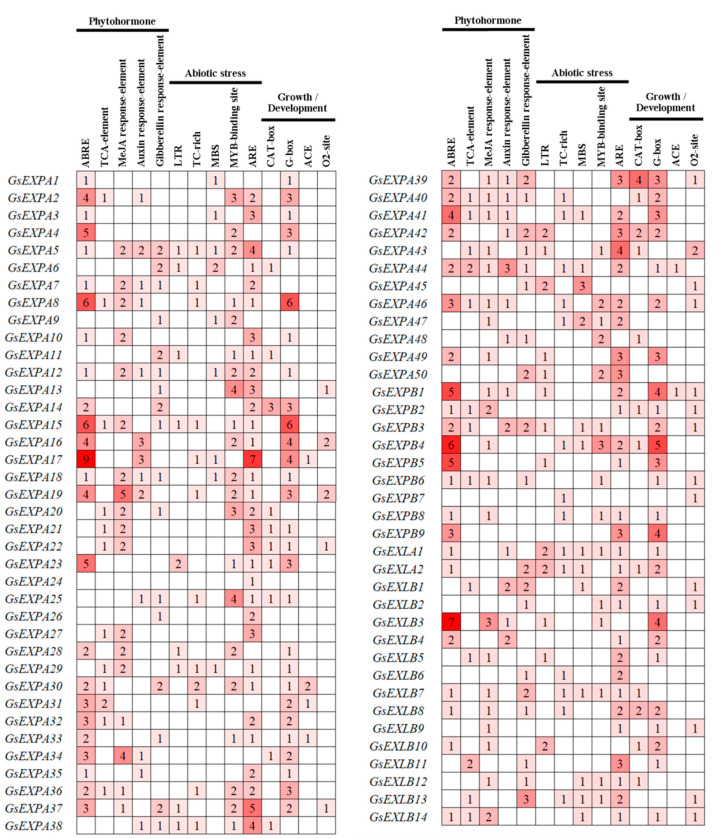
Promoter analysis results of wild soybean expansin family members.

We predicted cis-acting elements in the 1.5 kb promoter region of wild soybean expansin members by the PlantCARE online tool. The results are shown in Figure 4. These cis-acting elements mainly comprise three major classes: plant hormones, abiotic stresses and growth or development. Phytohormone response elements include ABRE (abscisic acid), TCA-element (salicylic acid), MeJA response-element (methyl jasmonate), auxin response-element and gibberellin response-element. Abiotic stress-responsive elements include LTR (low temperature response), TC-rich (defense and stress response), MBS (drought related), etc. Cis-acting elements associated with growth and development include CAT-box, G-box, ACE, etc. These results suggest that the expansin family may play a role in growth or development and response to environmental stress in wild soybean, and the transcription of genes may be regulated by phytohormones.

### 2.4. Wild Soybean Vegetative Organ Basal Transcriptome

**Figure 5:**
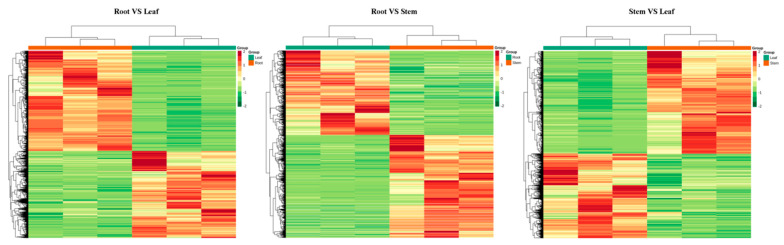
Cluster heat map of differential genes in the wild soybean vegetative organ transcriptome.

**Figure 6:**
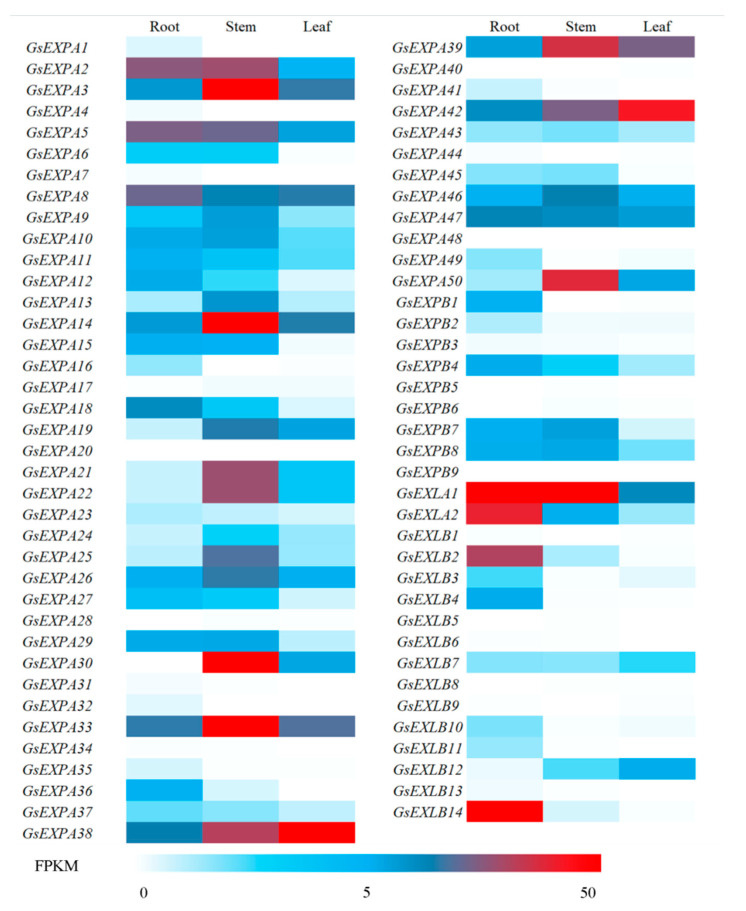
Heatmap of wild soybean expansins expression levels in vegetative organ transcriptome. FPKM, fragments per kilobase of transcript per million fragments mapped.

We measured the basal transcriptomes of three vegetative organs from 30 d wild soybean seedlings. Three replicates per organ yielded a total of 60.48 GB clean data, 6 GB of each sample, and percentage of Q30 bases at 88% and above. The number of differentially transcribed genes in the three organs analyzed was 19615, 13966, and 11523 for root to leaf, root to stem, and stem to leaf, respectively. The differential gene expression heatmap is shown in Figure 5. Building on the transcriptome data, we obtained expression quantity information for all wild soybean expansin members and plotted a heatmap (Figure 6). We found that the transcription of these genes has some organ specificity. Five of these genes were specifically expressed in the roots: *GsEXPA36*, *GsEXPB1*, *GsEXLB2*, *GsEXLB4*, and *GsEXLB14*. There were four stem-specific genes: *GsEXPA13*, *GsEXPA21*, *GsEXPA22* and *GsEXPA25*. No leaf-specific genes were found. There were also significant differences in transcript levels among expansin members, with the highest value being for *GsEXLA1* in roots (FPKM value average 233.58), whereas the transcript levels of *GsEXPA4*, *GsEXPA48*, *GsEXPB9*, and *GsEXLB5* were barely detectable.

### 2.5. Expression Profiles

**Figure 7:**
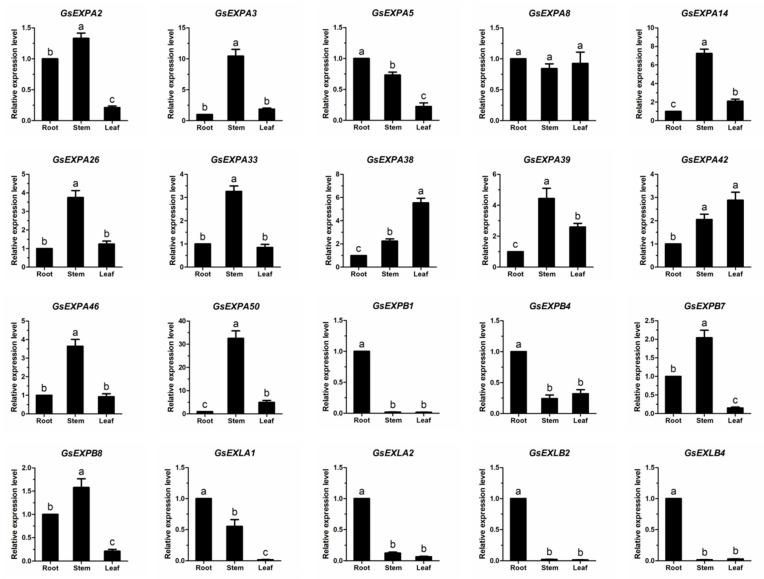
Expression levels of candidate 20 expansin genes detected by qRT-PCR in wild soybean root, stem, and leaf. Different small letters indicate that the transcription levels of expansin genes in different organs is significantly different (*p* < 0.05).

**Figure 8:**
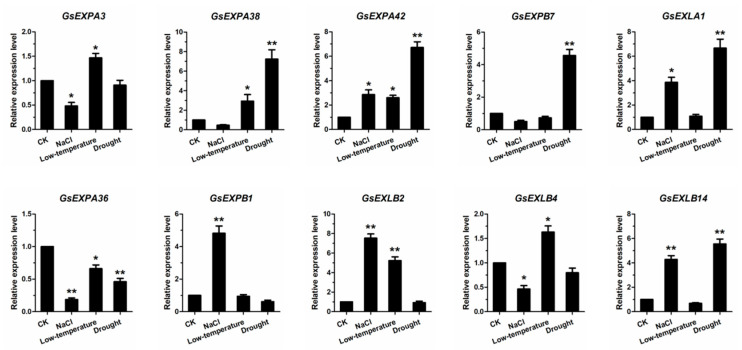
qRT-PCR detection of candidate 10 wild soybean expansin genes response to salt, low temperature and drought stress. * indicates that the transcription level of the expansin gene is significantly different from that of control group (*, *p* < 0.05; **, *p* < 0.01).

To further identify the expression patterns of expansin family members in vegetative organs, we examined the transcript levels of 20 candidate genes by qRT–PCR (Figure 7). The results showed that the transcription levels of these 20 genes were in good agreement with the transcriptome. In parallel, we examined the response of 10 candidate expansin genes to NaCl stress, low temperature, and drought treatments. These genes cover four subfamily members, including five root-specific genes (Figure 8). Among them, NaCl treatment significantly inhibited the transcription of *GsEXPA3*, *GsEXPA36*, and *GsEXLB4* but promoted the expression of *GsEXPA42*, *GsEXLA1*, *GsEXPB1*, *GsEXLB2*, and *GsEXLB14*. *GsEXPA3*, *GsEXPA38*, *GsEXPA42*, *GsEXPA36*, *GsEXLB2*, and *GsEXLB4* showed a stronger response to chilling stress at 4 °C. Meanwhile, the transcription levels of *GsEXPA38*, *GsEXPA42*, *GsEXPB7*, *GsEXLA1*, and *GsEXLB14* also showed a significant increase in response to drought stress, although *GsEXPA36* was more sensitive. Based on the above data, we selected *GsEXPB1*, which is specifically expressed in roots and actively responds to NaCl treatment, for further research.

### 2.6. Amino Acid Evolutionary Tree and Subcellular Localization of GsEXPB1

**Figure 9:**
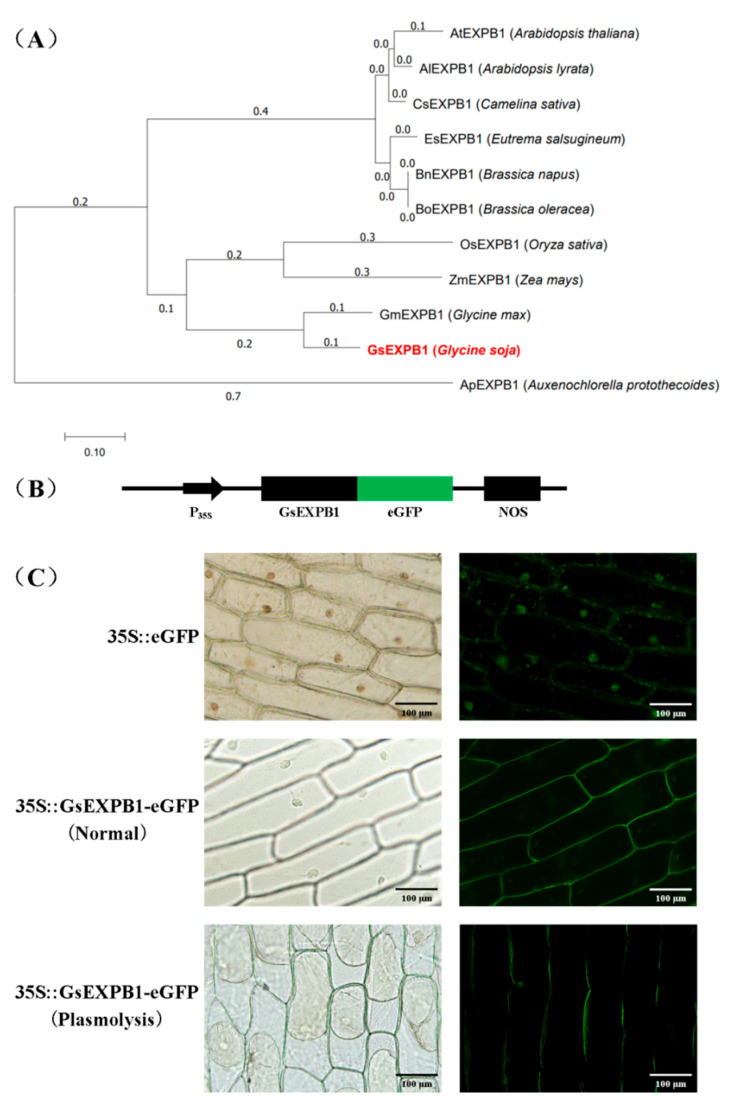
Amino acid phylogenetic tree of GsEXPB1 and results of its subcellular localization in onion epidermal cells. (**A**) Amino acid phylogenetic tree of GsEXPB1 and EXPB1s in other 10 plant species, values indicate evolutionary branch length (genetic variability). (**B**) GsEXPB1 expression vector construction for onion epidermal cells subcellular localization test. P_35S_ indicates CaMV35S promoter, eGFP indicates enhanced green fluorescent protein, NOS indicates terminator. (**C**) GsEXPB1 subcellular localization results, bar = 100 μm.

We selected 10 EXPB1 proteins that have been published in the NCBI database together with GsEXPB1 to construct an amino acid evolution tree. The results showed that GsEXPB1 was relatively closely related to GmEXPB1 homologs in soybean in the same branch (Figure 9A). In parallel, we performed subcellular localization of GsEXPB1 protein using onion epidermis as a model. Localization of GsEXPB1 to the cell wall in onion was demonstrated by laser confocal microscopy (Figure 9C).

### 2.7. Phenotypic Observation of Soybean Hairy Roots Overexpressing GsEXPB1

**Figure 10:**
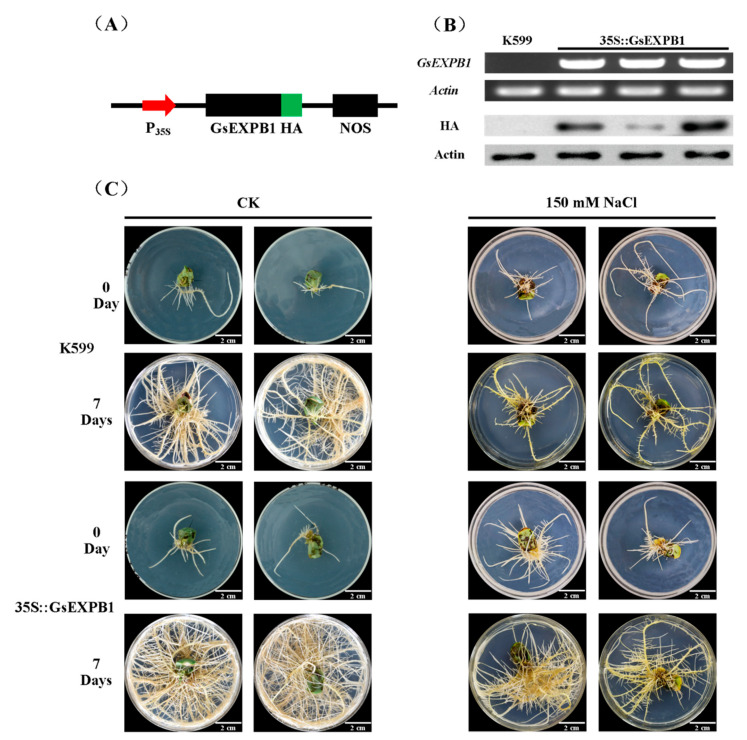
Overexpression of GsEXPB1 significantly promoted cultivated soybean hairy roots growth and salt stress tolerance. (**A**) Construction of expression vector, P_35S_ indicates CaMV35S promoter, HA indicates HA protein tag, NOS indicates terminator. (**B**) Detection results of gene transcription and protein expression in transgenic hairy roots. (**C**) Phenotypic observation of hairy roots overexpressing GsEXPB1 under normal growth conditions and salt stress, bar = 2 cm.

**Figure 11:**
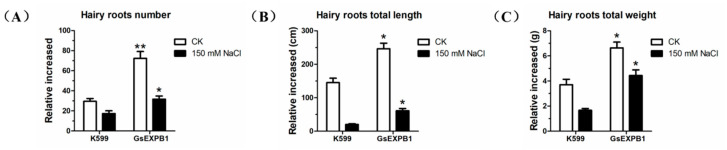
Phenotypic quantification results of soybean hairy roots overexpressed GsEXPB1. (**A**) Relative increase in the number of hairy roots. (**B**) Relative growth of total root length. (**C**) Relative increase in total root weight. * indicates a significant difference from K599 (*, *p* < 0.05; **, *p* < 0.01) (n = 50).

The *GsEXPB1* gene is specifically expressed in wild soybean roots and actively responds to NaCl treatment, for which we initially identified its function by a cultivated soybean hairy root transformation assay. RT–PCR and Western blot results demonstrated that this gene was already expressed (Figure 10B). We simultaneously calculated the transformation efficiency to be 93.33% (n = 50). Compared with the K599 control, transgenic hairy roots showed a better growth status under normal growth conditions (Figure 10C), and the relative growth of root number, total root length and root weight were all significantly improved (Figure 11). Under 150 mM NaCl stress, the growth of both control and transgenic hairy roots was significantly inhibited, but the latter was to a lesser extent. The number of hairy roots in the control group increased by only 17.33 at 7 d, whereas this value was 31.70 in transgenic roots. Meanwhile, the relative increases in total root length and root weight of transgenic plants were also enhanced by 2.05- and 1.65-fold, respectively, compared with the control group.

## 3. Discussion

### 3.1. Characterization of the Wild Soybean Expansin Family

Expansins are also required for plant growth and development because of their critical function in the process of plant cell wall relaxation. From successive reports of genome-wide identification of expansin, we know that members of this superfamily are generally numerous. For example, in wheat, moso bamboo, and *Gossypium hirsutum,* the numbers are 241, 82 and 93, respectively [8,44,45]. In wild soybean, this family is similarly large, with 75 members covering four subfamilies and the same number of members as in cultivated soybean [39]. This result also indicated a close evolutionary and genomic relationship between wild soybean and cultivated soybean. In terms of the subfamily member number, the EXPA family is more abundant in wild soybean, accounting for two thirds of all members, while the EXLA family has only two members. This is similar to the identification results in other plants, where EXPA family members usually account for most of the proportion (Table 1). During the analysis of wild soybean expansin gene structure, we found that the number of introns and conserved motifs was more similar among members within the same subfamily, also indicating that they are more conserved among members within the subfamily (Figure 2 and Figure 3). *GsEXPB5* has an extra-long intron and may have a special function, which we will target for further studies in the future.

Plant growth involves two major pathways, cell number increase and cell volume enlargement, while expansin functions in the latter process. In addition, the cell wall of plants is the first barrier to respond to and defend against external environmental stress, and alterations in the structure or composition of the cell wall are one of the mechanisms by which plants rapidly adapt to external stress, which in turn determines the ability of expansins to participate in the regulation of plant environmental stress resistance. Promoter prediction analysis results of wild soybean expansin members showed that the expression of these genes is correlated with plant growth and abiotic stress responses (Figure 4). Numerous studies have shown that the functions played by expansins in these two aspects are often positive, such as promoting plant root growth and increasing environmental stress capacity [32]. However, the opposite has also been reported. For example, tobacco overexpressing the *Populus tomentosa PtoEXPA12* gene had significantly less tolerance to Cd stress than the wild type [46]. Thus, there is still a dialectical desight of expansin function. qRT–PCR tests showed that the transcription of 10 candidate expansin genes responded differently to NaCl, low temperature, and drought stress (Figure 8). These results indicate that expansin has a large correlation with the strong environmental adaptation ability of wild soybean and can be used as a resistance gene resource for further exploration in subsequent work. Similar to findings in other plants, the transcription of wild soybean expansin genes may also be regulated by phytohormones, including ABA, SA, MeJA, and others. These phytohormones tend to be important components of expansin protein regulatory pathways. However, few studies have reported on the molecular regulatory mechanism of expansin at this stage, which is also an important part missing from related studies.

The expression of expansin genes in plants has some organ specificity. For example, *BdEXPA27* in *Brachypodium distachyon* [47], *OsEXPB5* in rice [48], and *HvEXPB1* in barley [14] are all specifically expressed in roots. In this study, we obtained transcriptional information for all genes in vegetative organs of wild soybean W05 by transcriptome technology. The results showed a large differential expression of these genes in roots, stems and leaves (Figure 5), and this was the same in the expansin family. More expansin genes were expressed in roots than in stems and leaves, and the highest number of specifically expressed genes was five in roots (Figure 6). However, in the present study, we only obtained transcriptome data for one growth period under normal growth conditions, thus having certain limitations for transcriptional pattern analysis of these genes in nutritional organs. Furthermore, the functions performed by the same gene in different organs of a plant are not exactly the same; thus, differences in its transcript levels do not represent the magnitude at which it exerts its effect. Among the multiple organs of plants, the root system is the main organ that responds to stresses such as salt and drought [49]. Although the data we obtained have certain limitations, these genes specifically expressed in roots may be involved in the progression of plant response to above stresses. Subsequent qRT–PCR test results also confirmed this speculation (Figure 8). At the aggregate level, expansin genes were expressed at higher transcript levels (FPKM value) in stems. This indicates that expansin is closely related to the growth of wild soybean stem. The stem of wild soybean is a coiled stem, which is quite different from the straight stem of cultivated soybean. Expansin may be involved in determining the growth habit of wild soybean stem.

### 3.2. Potential Functions of GsEXPB1

Regarding the action mechanism of the expansins, two hypotheses, “acid growth” and “nonenzymatic mechanism”, are commonly recognized [50,51]. However, whichever hypothesis is correct, the location where the expansins perform their functions is usually in the cell wall, and GsEXPB1 is also located in the cell wall of onion epidermal cells (Figure 9C). However, expansin proteins have also been reported to localize in the cell membrane, such as HvEXPB7 in barley [52] and OsEXPA17 in rice [53]. In the present study, the plant material used for subcellular localization was epidermal cells of onion with certain limitations. In subsequent functional studies on this protein, we will carefully select an appropriate system for the subcellular localization analysis. *GsEXPB1* was specifically expressed in roots and positively responded to NaCl treatment; thus, we preliminarily identified the function of this protein in cultivated soybean hairy root transformation. Expansins have the function of promoting plant root growth, which has been widely confirmed. In the present study, the growth status of soybean hairy roots overexpressing GsEXPB1 was significantly better than that of the control (Figure 10C), which was also supported by phenotypic quantification data (Figure 11).

Root growth involves cell division, elongation and other processes, whereas expansins are generally considered to perform functions during cell elongation. In the “acid growth” model, the pH-dependent increase in cell growth and wall extensibility occurs under many circumstances. A typical example is the process of auxin-mediated cell elongation. Auxin activates proton pumping across the plasma membrane, driving down extracellular pH, which in turn activates the expansins, facilitating yielding and stress relaxation of the stretched cell wall and leading to water uptake and cell enlargement. This scenario is typical of rapidly growing organs such as hypocotyls, where cells enlarge many-fold, filling up with a water-filled vacuole as they do so [54]. In this study, we used the plant material as hairy roots of soybean, which is also characterized by rapid growth and high water content. Increased expression of expansins should be expected to contribute significantly to cell wall relaxation. Whereas the cell wall serves as an important component to fix plant cell shape and restrict cell size, its excessive relaxation may increase water uptake by protoplasts or vacuoles, nearly rendering the cell larger. We speculate that this may be one of the reasons that GsEXPB1 is able to promote root growth. Based on the pilot data at this stage, we do not yet know whether the function exerted by GsEXPB1 has a direct effect on the increase in cell number in transgenic roots. We speculate that elongation of root cells by expansin may be auxin related and that this “acid growth” mediated by auxin may be maintained by more auxin accumulation. This auxin accumulation may have its origin in cotyledons, and in the growing vigorous root tip, auxin levels may be significantly increased. Increased auxin similarly induces more divisions in root cells, which in turn promotes elongation of transgenic roots. These will be explored in depth in subsequent functional and mechanistic studies on GsEXPB1. The well-developed root system is more conducive to the absorption of water and nutrients by plants and enhances the ability to cope with water and osmotic stress. Although our detailed mechanism by which GsEXPB1 functions remains unclear, this function of GsEXPB1 may have some applications for resistance breeding efforts in cultivated soybean.

Compared with wheat, rice, upland cotton and other crops, soybean is usually classified as a salt-sensitive crop, and its yield and quality will be significantly reduced under salt stress. With the increasing salinization of cultivated soils worldwide, it is of great value and significance to breed cultivated soybean cultivars that are salt tolerant. Most studies have shown that expansins similarly have positive functions in regulating salt stress tolerance in plants. For example, *NtEXPA4* in tobacco [55], *TaEXPA2* in wheat [30], *RhEXPA4* in rose [56], *OsEXPA7* in rice [31], etc. These expansins generally function in pathways such as increasing the ductility of the cell wall, reducing water loss, enhancing the viability of antioxidant enzyme lines and the content of osmoregulatory substances, and regulating Na^+^/K^+^ ion accumulation. In this study, the growth of soybean hairy roots overexpressing *GsEXPB1* under 150 mM NaCl stress, although somewhat inhibited, overall showed better tolerance to salt stress, indicating that it can be used as a candidate resistance gene for future studies. Upregulated expression of expansins in the face of water stress such as salt and drought is generally considered an adaptive response, enabling roots to continue growing despite reduced turgor pressure. This has the adaptive effect of increasing the root:shoot ratio, allowing roots to explore the soil for water while limiting the leaf surface area where water is lost to the atmosphere. This may also be one of the reasons that the growth status of overexpressing GsEXPB1 hairy roots was better than that of the control group under 150 mM NaCl stress. Our understanding of the mechanism by which *GsEXPB1* functions is also an important focus of related work at the next stage. Next, we will carry out salt stress-related functional and mechanistic studies of *GsEXPB1* using wild soybean and cultivated soybean as material systems, expecting to provide more quality gene resources for salt stress resistance improvement in cultivated soybean.

## 4. Materials and Methods

### 4.1. Genome-Wide Identification of Wild Soybean Expansin Genes

The most recent reference genome for wild soybean was derived from the NCBI database (https://www.ncbi.nlm.nih.gov/genome/?term=txid3848[orgn], accessed on 9 October 2020). A hidden Markov model (HMM) of expansin was built using conserved domains of DPBB_1 (PF03330) and Pollen_allerg_1 (PF01357) derived from the Pfam database (https://pfam.xfam.org/, accessed on 2 November 2020) and searched against the genome of wild soybean. Primary screening results were manually aligned by the NCBI-CDD database (NCBI Conserved Domain Database) and SMART database (Simple Modular Architecture Research Tool), while the results were aligned again and named combined with NCBI online blast results. The amino acid evolutionary tree of expansin family members was made by MEGA 7.0 software. Analysis of gene structure was performed online by GSDS 2.0 (https://gsds.gao-lab.org/, accessed on 12 November 2020), promoter cis-acting element prediction was performed online by PlantCARE (https://bioinformatics.psb.ugent.be/webtools/plantcare/html/, accessed on 15 November 2020), and the meme suite online software (https://meme-suite.org/meme/, accessed on 16 November 2020) was used to perform motif analysis.

### 4.2. Determination of the Basic Transcriptome in Nutritional Organs

Seeds of wild soybean W05 were provided by the Key Laboratory of Soybean Biology of the Chinese Education Ministry. Seeds were treated with 98% H_2_SO_4_ for 12 min to soften the seed coat and were sown in a nutrient mortar with vermiculite as a matrix. The culture conditions were as follows: 25/22 °C day/night temperature, 16/8 h light cycle, and watering with an appropriate amount of Hoagland nutrient solution daily. Samples were taken while seedlings were growing to Day 30 p.i. Root, stem, and leaf samples were snap frozen in liquid nitrogen and sent to Wuhan Maiteville Biotechnology Co., Ltd. (Hubei, China) for transcriptome sequencing. The fragment size and concentration of the libraries were detected using an Agilent 2100 Bioanalyzer. Thereafter, the library was sequenced using the Illumina HiSeq platform. Cuffquant and Cuffnorm use fragments per kilobase of transcript per million fragments mapped (FPKM) as an indicator of transcript or gene expression levels. The transcript levels of all members were determined according to the results of expansin genome-wide identification. Excel 2010 software was used to create a heatmap.

### 4.3. qRT–PCR

The transcript levels of 20 expansin genes in roots, stems, and leaves and the response of 10 candidate expansin genes to abiotic stress treatments were determined by qRT–PCR assays. We selected 20 genes with higher transcript levels (FPKM value ≥ 5) in each of the four subfamilies as targets, also considering whether these genes have specificity for organ expression. Cultivation methods and conditions of wild soybean were the same as above. For examination of the transcript levels of the 20 candidate genes in nutritional organs, we used roots, stems and leaves as plant materials, respectively. Stress treatments were as follows: salt stress was mimicked by 150 mM NaCl for 4 h, low temperature stress was 4 °C for 4 h, and drought stress was 12 d without watering. For the selection of 10 candidate expansin genes for abiotic stress treatments, results of promoter prediction analysis and the transcript levels in different nutritional organs were used as a reference, and four subfamily members were covered. The detection of transcript levels of *GsEXPA3*, *GsEXPA38*, *GsEXPA42*, *GsEXPB7* and *GsEXLA1* used the whole plant as the material, and *GsEXPA36*, *GsEXPB1*, *GsEXLB2*, *GsEXLB4* and *GsEXLB14* used roots as the sample.

For qRT–PCR, total RNA was extracted using a TIANGEN RNAprep Pure Plant Kit (Beijing, China), and cDNA was synthesized using TRANSGEN One-Step gDNA Removal and cDNA Synthesis SuperMix (Beijing, China). qRT–PCR assays were performed using TRANSGEN Top Green qPCR SuperMix (Beijing, China). The amplification of *Actin-11* (GenBank: LOC114395252) in wild soybean was used as an internal control. The expression levels for all candidate genes were determined using the 2^−ΔΔCT^ method, and relative transcript levels were calculated and normalized as described previously [57]. All the primers used in this research are listed in Supplementary Table S2.

### 4.4. Construction of the GsEXPB1 Amino Acid Evolutionary Tree and Its Subcellular Localization

Through pretests, we selected *GsEXPB1*, which is specifically expressed in roots and actively transcribed in response to salt stress, as the subject of further research. The published EXPB1 proteins from 10 other plants were used to construct an amino acid evolution tree of GsEXPB1. Subcellular localization of GsEXPB1 was determined by transient transfection in onion (*Allium cepa*) epidermal cells. Full-length *GsEXPB1* was obtained from the cDNA of wild soybean W05 by PCR. *GsEXPB1* was cloned into the pCambia1302::eGFP vector by homologous recombination to produce the GsEXPB1-eGFP fusion protein (Figure 9B). The 35S::eGFP and 35S::GsEXPB1-eGFP vectors were transformed into *Agrobacterium tumefaciens* GV3101 by the freeze–thaw method. Subcellular localization assays were performed according to the method of Chen et al. (2016) [58]. Transformed onion cells were observed using a confocal microscope (Olympus, Tokyo, Japan).

### 4.5. Overexpression of GsEXPB1 through Soybean Hairy Roots

*GsEXPB1* was ligated into the pCambia1302 vector by the homologous recombination method to overexpress HA-tagged protein (Figure 10A). The 35S::GsEXPB1-HA vector was transformed into *Agrobacterium rhizogenes* K599 by freeze thawing. Transformation of cultivated soybean hairy roots was performed with reference to Li et al. (2014) [59], and the variety of soybean was Dongnong 50, which was provided by the College of Agriculture, Northeast Agricultural University. The medium used for soybean hairy root induction was 1/2 MS solid medium (sucrose as carbon source, 30 g/L, agarose, 7 g/L). Culture conditions were a constant temperature of 28 °C and a light cycle of 16/8 h. RT–PCR and Western blotting were used to detect the transcription and protein expression of GsEXPB1, respectively. The procedures for RNA extraction and cDNA synthesis were as above. *Actin* (GenBank: LOC100777460) was selected as an internal reference gene for soybean. Expression of GsEXPB1 protein was detected by HA antibody, and actin was used as an internal reference.

### 4.6. Phenotypic Observation of Hairy Roots from Soybean Overexpressing GsEXPB1

Soybean hairy roots with positive test results were replanted into a new medium for cultivation. Photographs were taken on the first day and 7 d later, and relative increases in the number of hairy roots, total root length, and weight were determined and calculated. For salt stress treatment, NaCl was added to the culture medium to mimic salt stress at a concentration of 150 mM.

### 4.7. Statistical Analysis

All trials were repeated at least three times, and the data are shown as the mean ± standard deviation. Graphpad prism 5 software was used to perform significance analysis and plotting.

## 5. Conclusions

In this study, we systematically characterized the expansin family based on the genome of wild soybean W05 and obtained a total of 75 genes. The functions of these members may be relevant for the regulation of plant growth and development, abiotic stress, and phytohormone responses, and their transcription has obvious organ specificity. Meanwhile, we obtained an expansin gene, *GsEXPB1*, localized in the cell wall, specifically expressed in roots and responsive to salt stress. This protein has a certain positive role in promoting soybean root growth and improving salt stress tolerance.

## Figures and Tables

**Table 1:** Number of members in each subfamily of expansin in ten plant species.

Species (Scientific Name)	Total Number	EXPA	EXPB	EXLA	EXLB
*Gossypium hirsutum*	93	67	8	6	12
*Triticum aestivum*	241	121	104	16	0
*Moso Bamboo*	82	45	7	1	29
*Brachypodium distachyon*	38	30	4	3	1
*Gossypium hirsutum*	93	67	8	6	12
*Ziziphus jujuba*	30	19	3	1	7
*Glycine max*	75	49	9	2	15
*Nicotiana tabacum*	52	36	6	3	7
*Solanum lycopersicum*	38	25	8	1	4
*Glycine soja*	75	50	9	2	14

## Supplementary Materials

The following supporting information can be downloaded at: www.mdpi.com/article/10.3390/ijms23105407/s1.
