# Supplementary material for: Genome-Wide Identification of Expansin Genes in Wild Soybean (Glycine soja) and Functional Characterization of Expansin B1 (GsEXPB1) in Soybean Hair Root
Source: Int J Mol Sci. 2022 May 12;23(10):5407. doi: 10.3390/ijms23105407 (PMC9140629; doi:10.3390/ijms23105407)
Supplement: Supplementary file 1 [file ijms-23-05407-s001.zip › ijms-1711973-supplementary.pdf]

**Supplementary Table S1**

**Table S1.** Information of wild soybean expansin super family members

| Gene name | Gene locus | Protein length(aa) | Intron No. | Signal peptide(aa) | pI | Mw(kD) |
|---|---|---|---|---|---|---|
| *GsEXPA1* | LOC114370335 | 254 | 2 | 25 | 9.39 | 27.7 |
| *GsEXPA2* | LOC114372850 | 259 | 2 | 22 | 9.58 | 28.1 |
| *GsEXPA3* | LOC114372869 | 255 | 2 | 25 | 7.55 | 27.4 |
| *GsEXPA4* | LOC114374276 | 260 | 2 | 25 | 9.57 | 28.5 |
| *GsEXPA5* | LOC114376517 | 257 | 1 | 20 | 9.45 | 28.0 |
| *GsEXPA6* | LOC114379323 | 254 | 2 | 21 | 8.26 | 28.2 |
| *GsEXPA7* | LOC114379650 | 256 | 2 | 28 | 9.67 | 28.6 |
| *GsEXPA8* | LOC114380203 | 259 | 2 | 22 | 9.60 | 28.2 |
| *GsEXPA9* | LOC114381092 | 258 | 2 | 21 | 9.57 | 28.1 |
| *GsEXPA10* | LOC114383211 | 266 | 1 | 25 | 8.59 | 28.9 |
| *GsEXPA11* | LOC114383245 | 260 | 1 | 23 | 9.50 | 28.1 |
| *GsEXPA12* | LOC114383680 | 258 | 2 | 26 | 9.20 | 27.8 |
| *GsEXPA13* | LOC114384276 | 254 | 2 | 27 | 7.61 | 27.3 |
| *GsEXPA14* | LOC114386648 | 250 | 2 | 20 | 9.33 | 26.9 |
| *GsEXPA15* | LOC114387341 | 256 | 2 | 19 | 9.43 | 27.8 |
| *GsEXPA16* | LOC114391831 | 265 | 2 | 31 | 8.85 | 28.7 |
| *GsEXPA17* | LOC114392393 | 267 | 3 | 39 | 8.41 | 28.8 |
| *GsEXPA18* | LOC114392564 | 255 | 2 | 23 | 9.12 | 27.5 |
| *GsEXPA19* | LOC114394768 | 316 | 2 | None | 9.71 | 34.3 |
| *GsEXPA20* | LOC114394777 | 255 | 1 | 26 | 8.54 | 28.4 |
| *GsEXPA21* | LOC114395314 | 265 | 3 | None | 9.51 | 29.1 |
| *GsEXPA22* | LOC114395314 | 241 | 2 | 19 | 9.28 | 26.1 |
| *GsEXPA23* | LOC114395722 | 281 | 1 | 23 | 5.52 | 30.2 |
| *GsEXPA24* | LOC114395742 | 262 | 2 | 25 | 9.72 | 28.6 |
| *GsEXPA25* | LOC114397255 | 254 | 2 | 27 | 8.44 | 27.2 |
| *GsEXPA26* | LOC114397606 | 258 | 2 | 21 | 9.74 | 27.7 |
| *GsEXPA27* | LOC114397736 | 270 | 1 | 22 | 8.09 | 29.2 |
| *GsEXPA28* | LOC114398438 | 279 | 2 | 20 | 9.32 | 30.8 |
| *GsEXPA29* | LOC114398449 | 257 | 1 | 20 | 9.48 | 27.6 |
| *GsEXPA30* | LOC114399108 | 250 | 3 | 25 | 9.42 | 27.4 |
| *GsEXPA31* | LOC114399718 | 254 | 2 | 24 | 9.80 | 27.9 |
| *GsEXPA32* | LOC114402569 | 254 | 2 | 25 | 9.40 | 27.8 |
| *GsEXPA33* | LOC114403690 | 248 | 2 | 20 | 9.32 | 26.6 |
| *GsEXPA34* | LOC114405974 | 249 | 2 | 24 | 9.55 | 26.8 |
| *GsEXPA35* | LOC114407490 | 255 | 2 | 26 | 9.55 | 27.8 |
| *GsEXPA36* | LOC114408550 | 256 | 2 | 24 | 8.63 | 27.6 |
| *GsEXPA37* | LOC114409709 | 248 | 2 | 20 | 8.96 | 26.7 |
| *GsEXPA38* | LOC114410169 | 250 | 2 | 22 | 8.37 | 26.8 |
| *GsEXPA39* | LOC114411409 | 250 | 2 | 20 | 9.33 | 26.9 |

| | | | | | | |
|---|---|---|---|---|---|---|
| *GsEXPA40* | LOC114414291 | 252 | 2 | 18 | 8.97 | 27.3 |
| *GsEXPA41* | LOC114414569 | 255 | 2 | 23 | 8.75 | 27.4 |
| *GsEXPA42* | LOC114415692 | 250 | 2 | 22 | 8.37 | 26.8 |
| *GsEXPA43* | LOC114416556 | 249 | 2 | 21 | 9.11 | 26.7 |
| *GsEXPA44* | LOC114416888 | 257 | 2 | 21 | 9.55 | 28.5 |
| *GsEXPA45* | LOC114417223 | 254 | 2 | 21 | 8.01 | 28.3 |
| *GsEXPA46* | LOC114418935 | 258 | 2 | 21 | 9.76 | 27.8 |
| *GsEXPA47* | LOC114419868 | 248 | 2 | 20 | 9.34 | 26.5 |
| *GsEXPA48* | LOC114422475 | 259 | 1 | 26 | 8.05 | 28.8 |
| *GsEXPA49* | LOC114424245 | 260 | 2 | 25 | 9.44 | 28.6 |
| *GsEXPA50* | LOC114425810 | 265 | 3 | None | 9.51 | 29.1 |
| *GsEXPB1* | LOC114370793 | 277 | 3 | 29 | 5.43 | 29.5 |
| *GsEXPB2* | LOC114375549 | 277 | 3 | 29 | 4.95 | 29.4 |
| *GsEXPB3* | LOC114375883 | 258 | 3 | 23 | 9.11 | 28.2 |
| *GsEXPB4* | LOC114379096 | 267 | 3 | 31 | 8.66 | 29.1 |
| *GsEXPB5* | LOC114401963 | 155 | 2 | 29 | 4.94 | 16.7 |
| *GsEXPB6* | LOC114405382 | 267 | 3 | 24 | 5.37 | 28.7 |
| *GsEXPB7* | LOC114413024 | 277 | 3 | 29 | 4.87 | 29.5 |
| *GsEXPB8* | LOC114417222 | 267 | 3 | 31 | 8.66 | 29.2 |
| *GsEXPB9* | LOC114369208 | 272 | 3 | 29 | 7.51 | 29.1 |
| *GsEXLA1* | LOC114377455 | 259 | 4 | 19 | 8.28 | 28.0 |
| *GsEXLA2* | LOC114378544 | 261 | 4 | 19 | 8.47 | 28.3 |
| *GsEXLB1* | LOC114373430 | 272 | 4 | 38 | 5.06 | 29.9 |
| *GsEXLB2* | LOC114374907 | 251 | 3 | 25 | 5.51 | 28.2 |
| *GsEXLB3* | LOC114392387 | 247 | 3 | 24 | 6.30 | 27.7 |
| *GsEXLB4* | LOC114392841 | 251 | 3 | 24 | 5.42 | 27.9 |
| *GsEXLB5* | LOC114392916 | 248 | 3 | 24 | 9.16 | 27.4 |
| *GsEXLB6* | LOC114392956 | 250 | 3 | 24 | 9.35 | 27.5 |
| *GsEXLB7* | LOC114392974 | 251 | 4 | 21 | 4.86 | 27.2 |
| *GsEXLB8* | LOC114411512 | 241 | 3 | 24 | 8.78 | 26.8 |
| *GsEXLB9* | LOC114412218 | 244 | 3 | 24 | 8.98 | 27.1 |
| *GsEXLB10* | LOC114412220 | 247 | 3 | 24 | 8.31 | 27.6 |
| *GsEXLB11* | LOC114412221 | 251 | 3 | 24 | 5.60 | 28.0 |
| *GsEXLB12* | LOC114412251 | 250 | 4 | 20 | 5.13 | 27.0 |
| *GsEXLB13* | LOC114423099 | 255 | 4 | 21 | 4.49 | 27.8 |
| *GsEXLB14* | LOC114423317 | 251 | 3 | 24 | 5.32 | 28.3 |

**Supplementary Table S2**

**Table S2.** Primers used in this research

| Name | Sequence (5'-3') | Length (bp) | Purpose |
|---|---|---|---|
| GsEXPA2-FW | AGTGCTCACGCCACCTTCT | 19 | qRT-PCR |
| GsEXPA2-RV | ACCACTGCCTGTCGTTTGC | 19 | |
| GsEXPA3-FW | CTTTGATATGGCTGAACCTGC | 25 | qRT-PCR |
| GsEXPA3-RV | CCACCCAGTTTTGGACCC | 18 | |
| GsEXPA5-FW | CAGCCCTTCCATCTTCATCA | 20 | qRT-PCR |
| GsEXPA5-RV | AAGTGAGGGCGAGGAGGGTT | 20 | |
| GsEXPA8-FW | AGTGCCCACGCCACCTTCTA | 20 | qRT-PCR |
| GsEXPA8-RV | ACCACTGCCTGTCGTTTGC | 19 | |
| GsEXPA14-FW | TCTCAACCTGTTTTCCTTCG | 20 | qRT-PCR |
| GsEXPA14-RV | GGACCAAGTTGAAGTAGGAGTG | 22 | |
| GsEXPA26-FW | TGCCTCTGGAACAATGGGTG | 20 | qRT-PCR |
| GsEXPA26-RV | CTTGGTCGCACTTTATCTCG | 20 | |
| GsEXPA33-FW | CCACCTTCTATGGAGGGAGT | 20 | qRT-PCR |
| GsEXPA33-RV | AATCTCATAGCAAGAGCCACA | 21 | |
| GsEXPA36-FW | TGTGGGGAGTGTTACAAAATCA | 22 | qRT-PCR |
| GsEXPA36-RV | CATCCTCCGTTGTTGTTGG | 19 | |
| GsEXPA38-FW | TGCTGACTATGGAGGTGGATG | 21 | qRT-PCR |
| GsEXPA38-RV | GCAGTGACGATTATGCTACCG | 21 | |
| GsEXPA39-FW | GCCTCCCTGGCTCCATTAT | 19 | qRT-PCR |
| GsEXPA39-RV | CAATGCGAAGGAAGACAGG | 19 | |
| GsEXPA42-FW | TGGTTGGCAAGGTGGTCAT | 19 | qRT-PCR |
| GsEXPA42-RV | GGCAGAAGTTTGTGGCAGTAA | 21 | |
| GsEXPA46-FW | CGCCCTTCCGAACGACAAT | 19 | qRT-PCR |
| GsEXPA46-RV | TATGCGACGGGGACGATTC | 19 | |
| GsEXPA50-FW | TGGCTCTTCTTGGATTTCTTC | 21 | qRT-PCR |
| GsEXPA50-RV | ACCACAAGCCCCACCCATT | 19 | |
| GsEXPB1-FW | TTGTTTGAATCAGGCGAAGG | 20 | qRT-PCR |
| GsEXPB1-RV | TTTGCCAGCATTGCGTAGC | 19 | |
| GsEXPB4-FW | CTCGCCTTGAAACTCGTGC | 19 | qRT-PCR |
| GsEXPB4-RV | CGGCTTCACATCCACCATC | 19 | |
| GsEXPB7-FW | GGCAACCCCGTGAAAGTAG | 19 | qRT-PCR |
| GsEXPB7-RV | CTCATATTCAACCAAGGTAGCAAA | 24 | |
| GsEXPB8-FW | TGGGTATGGAACAATGGTGG | 20 | qRT-PCR |
| GsEXPB8-RV | CGGGGCACTCATCCGTAAT | 19 | |
| GsEXLA1-FW | GGTTGTGGTGCCTGCTTTC | 19 | qRT-PCR |
| GsEXLA1-RV | CAGGCTTCTTGCTTGATTCTT | 21 | |
| GsEXLA2-FW | CAAGGCTTCCTATTTCTCCAA | 21 | qRT-PCR |
| GsEXLA2-RV | CACCACAAACTGCTCCATCT | 20 | |
| GsEXLB2-FW | CTCTATCTTGGTGGAACCTACG | 22 | qRT-PCR |
| GsEXLB2-RV | TGAAACCTCAACTTTATGTCGC | 22 | |

| | | | |
|---|---|---|---|
| GsEXLB4-FW | ATAGCAACCCACCAAACGG | 19 | qRT-PCR |
| GsEXLB4-RV | GTGCCTATCTATTTCTAATCCGTTC | 25 | |
| GsEXPB1-FW-2 | TTATGGCTCCTACACTTCAACG | 22 | Gene clone |
| GsEXPB1-RV-2 | AAACAAATGGACAACTTCGATTA | 23 | |
